# Scoliosis

**Published:** 1888-12

**Authors:** 


### Translations.

IN ABSTRACT.

---

### *SCOLIOSIS.*

Its treatment has received new impetus from Strassburg by Ernst Fischer and H. Walferman. The first has long ago proven, through examinations of the skeleton of scoliotics, that the rotation is the primary factor in scoliosis and the shaping of a figure S secondary. Led by this principle, he introduced an exceedingly simple treatment, essentially consisting in torsion of the thorax by heavy weights (forty to 160 pounds) carried actively on the side where the ribs are prominent in the back, for about half an hour daily. At the start, of course, weight and time are lesser. To prevent slipping of the weight, it is attached to an elastic belt wound a couple of times around the chest. The patient leans the elbows on a chair, or holds in each hand a short crutch, and walks around the room in quadruped fashion. Fischer's results are, according to his numerous plates, most extraordinary. W. obtains similar results by a brace, which consists of two halves—one encircling the pelvis, the other the chest. They have to be made from plaster-paris casts and admit torsion against each other by a spiral spring, which acts continually. Luecke testifies to the good effects of the brace, which is patented in Germany. The reporter deems the active muscular exercise of Fischer's simpler, more effective, certainly more physiological, and more amply proven.

M. H.

For details, vide *Berliner Klinische Wochenschrift*, Nos. 39 and 40, and *Centralblatt für Chirurgie*, No. 42, 1888.

